# Physical Activity and Blood Lipids and Lipoproteins in Dialysis Patients

**DOI:** 10.1155/2012/106914

**Published:** 2012-09-18

**Authors:** Hiroyuki Imamura, Keiko Mizuuchi, Reika Oshikata

**Affiliations:** ^1^Department of Health and Nutrition, Faculty of Health Management, Nagasaki International University, 2825-7 Huis Ten Bosch, Sasebo-shi, Nagasaki 859-3298, Japan; ^2^School of Nursing, Fukuyama Heisei University, 117-1 Masado Kamiiwanari, Miyuki-cho, Fukuyama-shi, Hiroshima 720-0001, Japan; ^3^Faculty of Life Sciences, Seika Women's Junior College, 2-12-1 Minami Hachiman-cho, Hakata-ku, Fukuoka 812-0886, Japan

## Abstract

The relationship between physical activity and blood lipids and lipoproteins in dialysis patients is reviewed in the context of the potentially confounding factors such as nutritional intake, cigarette smoking, obesity, alcohol intake, and physical activity levels in the general population and additional confounding factors such as mode of dialysis and diabetes in dialysis patients. The known associations in the general population of physical activity with high-density-lipoprotein cholesterol subfractions and apolipoprotein A-I are more pronounced in hemodialysis patients than in peritoneal dialysis patients even after adjusting for these confounding factors. Examining studies on the effects of physical activity on blood lipids and lipoproteins, the most consistent observation is the noted decrease in triglycerides and increase in high-density-lipoprotein cholesterol and insulin sensitivity in hemodialysis patients. The changes in lipids and lipoproteins in hemodialysis patients could be caused by changes in activity levels of lipoprotein lipase, insulin sensitivity, and/or glucose metabolism. Future research investigating the relationship between physical activity and blood lipids and lipoproteins in dialysis patients should direct research towards the underlying mechanisms for changes in blood lipids and lipoproteins.

## 1. Introduction

Atherosclerotic heart disease is the leading cause of mortality among patients with chronic kidney disease [1–3]. Chronic kidney disease is associated with dyslipidemia, which seems to persist as renal failure advances and continues to affect clinical outcomes in patients on hemodialysis (HD) and peritoneal dialysis (PD) [4–13]. Patients on HD and PD are at increased risk for atherosclerotic heart disease, which is due at least in part to atherogenic lipid and lipoprotein abnormalities [1]. One study [14] compared traditional atherosclerotic heart disease risk factors among new dialysis patients with those in the general population and reported that the dialysis patients had a high prevalence of diabetes, hypertension, low physical activity, low high-density-lipoprotein cholesterol (HDL-C), and high triglycerides (TG). Exercise capacity as measured by maximal oxygen uptake in HD and PD patients is lower than in sedentary normal controls [15], but dialysis patients regardless of the treatment mode could benefit from appropriate exercise training in order to increase physical working capacity [16–18]. The positive association of physical activity with HDL-C has been reported in the general population [19–21]. Although a number of studies have reported blood lipid and lipoprotein profiles of HD and PD patients [5–13, 22], very little is known about how physical activity is related to or affects lipid and lipoprotein abnormalities in dialysis patients [22–25]. The purpose of this study was to review the scientific literature concerning the relationship between physical activity and blood lipids and lipoproteins in dialysis patients.

## 2. Confounding Factors in the General Population

When evaluating the predictive power of a certain risk factor, a variation in the factor can be influenced by a simultaneous variation in other possible confounding factors. Thus, it is important to adjust other confounding factors to examine the relationship of physical activity with blood lipids and lipoproteins.

### 2.1. Nutrition

It has been suggested that higher intake of n-3 polyunsaturated fatty acids and lower intakes of n-6 polyunsaturated fatty acids and saturated fatty acids have preventive effects on coronary heart disease [26–30]. Saturated fatty acids, cholesterol, and excess caloric intake raise serum low-density-lipoprotein cholesterol (LDL-C) [31]. Consumption of fruit and vegetables is inversely related to LDL-C [32–34]. In addition, individuals consuming a high-carbohydrate diet tend to show lower HDL-C than those who consume a low-carbohydrate diet [35, 36].

### 2.2. Cigarette Smoking

In studies at our laboratory [37–40], we reported that cigarette smoking was negatively associated with HDL-C [37] and positively with TG [37–40]. Cigarette smokers show significantly higher TG and lower HDL-C and/or HDL_2_-C [37–40].

### 2.3. Obesity

Obese subjects, in comparison with their counterparts, tend to show higher TC, TG, and LDL-C and/or lower HDL-C [41–46]. It has been reported that the percentage of body fat showed a significant positive correlation with TG and a negative correlation with HDL-C after adjusting for age and maximal oxygen uptake [47–49]. It has also been reported that body mass index (BMI) was positively related to LDL-C and TG [50].

### 2.4. Alcohol Intake

It has been reported that alcohol consumption was positively associated with HDL, HDL_2_, and/or HDL_3_ [51–53]. Alcohol drinkers show a higher HDL-C than nondrinkers [54–58].

## 3. Confounding Factors in Dialysis Patients

The relationships of confounding factors with blood lipids and lipoproteins in dialysis patients have not been investigated sufficiently in contrast to the above studies on the general population.

### 3.1. Mode of Dialysis

Both HD and PD patients exhibit a more atherogenic lipid profile, such as increased serum TG, and decreased apolipoprotein (apo) A and/or HDL-C [5–13] than healthy controls. However, PD patients exhibit a more atherogenic lipid profile than HD patients [10, 11, 13, 22]. The cause of the worsening of lipid profile in PD patients may be multifactorial. First, PD patients have increased lipoprotein substrate availability through glucose uptake from the peritoneal dialysis fluid, which may contribute to increased hepatic synthesis of apo B-containing lipoproteins [59, 60]. Second, it may be related to the loss of large molecular weight substances in the peritoneal dialysis fluid [59, 60]. The daily clearance of apo A-I has been reported to be twofold to fourfold greater than that of apo B [61, 62]. Third, it has been reported that the PD patients had significantly higher apo CIII than HD patients [11], which may suggest that removal of TG-rich lipoproteins may be less efficient in the PD patients than HD patients because apo CIII inhibits lipoprotein lipase (LPL). LPL hydrolyzes both chylomicron and VLDL on the vascular endothelium and generates precursor of HDL during lipolysis of TG-rich lipoproteins [63]. Thus, decrease in this enzyme activity may decrease HDL-C and increase TG.

### 3.2. Diabetes

Sakurai et al. [8] compared lipid, apoprotein, and associated enzyme activities in 5 groups of subjects: nondiabetic end-stage renal disease (ESRD) patients with HD, nondiabetic ESRD patients without HD, diabetic ESRD patients with HD, diabetic ESRD patients without HD, and normal controls. The results showed that the 2 groups of nondiabetic ESRD with or without HD exhibited significantly higher serum TG and apo C-III and lower HDL-C, apo A-I, apo A-II, apo E, and LCAT activities than the controls. LPL activity was significantly lower in undialysed patient groups than the controls, whereas such differences were not found in dialysed patients groups. Hepatic triglyceride lipase activity was decreased in all 4 patient groups compared with that in controls. Patients with diabetic ESRD exhibited significantly higher serum apo B than controls, besides the lipid and apo abnormalities observed in nondiabetic ESRD patients. The apo B/apo A-I ratio was significantly higher in diabetic ESRD patient groups than in nondiabetic patient groups undergoing HD. These results indicate that lipid abnormalities are accelerated in diabetic ESRD patients.

## 4. Relationship of Physical Activity with Blood Lipids and Lipoproteins

### 4.1. General Population

It has been reported that physical activity was positively associated with HDL-C [19–21]. Active people in comparison with sedentary people tend to show lower TC and TG and/or higher HDL-C [64–67].

### 4.2. Dialysis Patients

In a study at our laboratory [21], we reported the relationship of physical activity, as measured by steps/day, with HDL-C subfractions and LCAT activity in 35 HD and 26 PD patients. In this study, nutrient and dietary intake, proportions of drinkers, smokers, and men and women did not differ between HD and PD patient groups. Furthermore, these variables did not correlate with any of the serum lipids, lipoproteins, and LCAT activity. Thus, influences of these variables appear to be limited. However, HD patients had significantly higher mean age and duration of dialysis treatment and had lower mean BMI and steps/day than PD patients, so that analysis was performed separately for HD and PD by computer. The results showed that, when possible confounding factors (LCAT activity, logarithmic transformation of TG, age, and gender) were included in the stepwise multiple regression analyses, in HD patients, steps/day was significantly positively correlated with HDL_2_-C and apo A-I, while it was significantly positively correlated with HDL_3_-C in PD patients. When subjects were subdivided into 3 groups according to steps/day, in HD patients, the highest category of steps/day had significantly higher HDL_2_-C and apo A-I than the lowest category, while such results were not observed in PD patients. These results suggest that the known associations in the general population of physical activity with HDL-C subfractions and apo A-I are more pronounced in HD patients than in PD patients. However, it needs to be mentioned that because the sample size is small in this study, further studies are needed to make firm conclusion.

## 5. Effects of Physical Exercise on Blood Lipids and Lipoproteins

### 5.1. General Population

It has been reported that aerobic training increases HDL-C [68, 69] and lowers TG, LDL-C, and/or VLDL-C [69, 70].

### 5.2. Hemodialysis Patients

Goldberg et al., using 6 [23] or 7 [24] subjects, examined the metabolic effects of exercise training in HD patients and reported that training lowered TG and increased HDL-C. In a subsequent study, Goldberg et al. [25] examined the effects of exercise on plasma lipids and lipoproteins in 25 HD patients. Patients were randomized into comparable exercising (*n* = 14) and sedentary control (*n* = 11) groups. All training sessions were held indoors, 3 times weekly for a mean of 12 ± 4 (SD) months, on a 17 lap/mile track. The results showed that maximal oxygen uptake significantly increased 21%, and the durations for the graded exercise stress test significantly improved 19% in exercising group, but did not change in controls. Plasma TG significantly decreased 37%. No change in TC or LDL-C was noted; however, HDL-C significantly increased 17% in the exercising group. Furthermore, fasting plasma insulin significantly decreased 20% and glucose disappearance rates significantly improved 42%. Such changes were not observed in the control group.

## 6. Mechanisms for Changes in Blood Lipids and Lipoproteins in Dialysis Patients

Several possible mechanisms that could explain how physical activity changes blood lipids and lipoproteins in dialysis patients have been proposed by Goldberg et al. [23]. First, they could be caused by changes in activity levels of LPL. Kantor et al. [71], using 10 well-trained men, reported that LPL activity was nearly doubled after participating in a marathon and suggested that increase in LPL activity probably mediated the increase in HDL-C. However, the relationship of physical activity with LPL activity has never been investigated in dialysis patients.

Second, it is also possible that they could be indirectly and/or directly caused by improvements of insulin sensitivity and glucose metabolism [23]. The elevation in plasma insulin may contribute to the lipid abnormalities observed in dialysis patients by promoting the synthesis and production of TG-rich lipoproteins by the liver. Goldberg et al. [23], using 6 HD patients, examined the metabolic effects of exercise training and reported that there was a 23% improvement in glucose tolerance and a 40% reduction in hyperinsulinism with no significant changes in body weight or diet.

## 7. Directions for Future Research

It is suggested that, in dealing with hyperlipoproteinemia, the first principle is the provision of a diet [72, 73] and the maintenance of a good exercise and physical fitness program. However, the relationships of physical activity with blood lipids and lipoproteins and effects of exercise training on dialysis patients have not been investigated sufficiently in contrast to that in the general population. For HD patients, it is suggested that the intensity of exercise should be between 65% and 85% of maximal levels, as determined from exercise testing. Interval work varying the exercise intensity between “target” intensity and lower for durations of 5 minutes may be necessary initially. The goal of 30 to 45 minutes of exercise at target intensity per session, 3 sessions per week, should be attained [74].

Future research investigating the relationship between physical activity and blood lipids and lipoproteins in dialysis patients should include (i) studies designed to investigate the relationships of physical activity with blood lipids and lipoproteins after adjusting for other possible confounding factors, (ii) effects of exercise training on blood lipids and lipoproteins, and (iii) directing research towards the underlying mechanisms for changes in blood lipids and lipoproteins. These studies should, in particular, focus on adolescents and young adults since there is a paucity of information in the literature in this area.
